# High triglyceride-glucose (TyG) index is associated with poor prognosis of heart failure with preserved ejection fraction

**DOI:** 10.1186/s12933-023-02001-4

**Published:** 2023-09-29

**Authors:** Qing Zhou, Jie Yang, Hongyi Tang, Zexuan Guo, Wenyue Dong, Yiting Wang, Xiangbin Meng, Kuo Zhang, Wenyao Wang, Chunli Shao, Xinwei Hua, Yi-Da Tang

**Affiliations:** 1 Department of Cardiology, State Key Laboratory of Cardiovascular Disease, Graduate School of Peking Union Medical College, National Center for Cardiovascular Diseases, Fuwai Hospital, Chinese Academy of Medical Sciences and Peking Union Medical College, Beijing, 100037 China; 2 Department of Cardiology, Institute of Vascular Medicine, State Key Laboratory of Vascular Homeostasis and Remodeling, NHC Key Laboratory of Cardiovascular Molecular Biology and Regulatory Peptides, Beijing Key Laboratory of Cardiovascular Receptors Research, Peking University Third Hospital, Peking University, Peking University, Beijing, 100191 China; 3 Department of Orthodontics, Peking University School and Hospital of Stomatology, Beijing, 100034 China; 4 School of Basic Medical Sciences, Peking University, Beijing, 100091 China

**Keywords:** Heart failure with preserved ejection fraction, Triglyceride-glucose index, Insulin resistance, Mortality

## Abstract

**Background:**

The impact of insulin resistance on the prognosis of heart failure with preserved ejection fraction (HFpEF) remains unknown. This study aimed to investigate the association between the triglyceride-glucose (TyG) index, an easily calculated marker of insulin resistance, and the long-term prognosis of HFpEF.

**Methods:**

A total of 823 patients with HFpEF were enrolled in the study. The TyG index was determined using the formula ln(fasting triglycerides [mg/dL] × fasting glucose [mg/dL]/2). The primary endpoint was all-cause death. The secondary endpoints were cardiovascular (CV) death and heart failure (HF) rehospitalization. Restricted cubic spline, multivariate Cox proportional hazard models, and competing risk models were used for analyses.

**Results:**

During a median follow-up period of 3.16 years, 147 (17.8%) all-cause deaths, 139 (16.8%) CV deaths, and 222 (27.0%) HF rehospitalizations occurred. Restricted cubic spline analysis revealed a J-shaped association between the TyG index and the mortality and rehospitalization rates. In the multivariate Cox proportional hazard models, compared with those in the lowest TyG index tertile, patients in the highest tertile exhibited the greatest susceptibility to all-cause death (HR 1.53, 95% CI 1.19–1.98) and CV death (HR 1.52, 95% CI 1.19–1.96). In the competing risk model, a significant association between the TyG index and HF rehospitalization was observed (HR 1.31, 95% CI, 1.07–1.61).

**Conclusion:**

A high TyG index is associated with an increased risk of mortality and rehospitalization in patients with HFpEF. The TyG index may serve as a promising prognostic marker for patients with HFpEF.

**Supplementary Information:**

The online version contains supplementary material available at 10.1186/s12933-023-02001-4.

## Introduction

Heart failure with preserved ejection fraction (HFpEF) is a prevalent and challenging clinical syndrome characterized by diastolic dysfunction and elevated filling pressures [1]. Despite advances in treatment, patients with HFpEF continue to experience high mortality rates and frequent rehospitalization, leading to a substantial healthcare burden [2]. Identification of novel risk factors associated with adverse outcomes in HFpEF is essential for improving patient care and prognosis.

Metabolic disturbances, including insulin resistance (IR), dyslipidemia, and abnormal fatty acid metabolism, have been implicated in HFpEF pathogenesis and progression [3, 4]. The triglyceride-glucose (TyG) index, a surrogate marker of IR, has emerged as a potential predictor of adverse cardiovascular (CV) outcomes in various populations [5–9]. The TyG index is calculated as the logarithm of the product of fasting plasma triglyceride (TG) and fasting plasma glucose (FPG) levels, reflecting the interplay between lipid and glucose metabolisms [5].

While previous studies have examined the association between the TyG index and CV outcomes in various populations, limited data are available regarding its impact on long-term prognosis, specifically in individuals with HFpEF. Furthermore, exploring the relationship between the TyG index and HFpEF established risk scores, such as the HFA-PEFF [10] and H_2_FPEF scores [11], could provide valuable insights into the prognostic utility of this metabolic marker within the existing risk assessment frameworks.

Therefore, this cohort study aimed to investigate the impact of the TyG index on the mortality and rehospitalization rates in patients diagnosed with HFpEF over a 3-year follow-up period. Additionally, we examined the relationship between TyG index across and HFA-PEFF and H_2_FPEF scores to provide valuable insights into the potential utility of the TyG index as a risk-stratification tool.

## Methods

### Study design and population

**Fig. 1:**
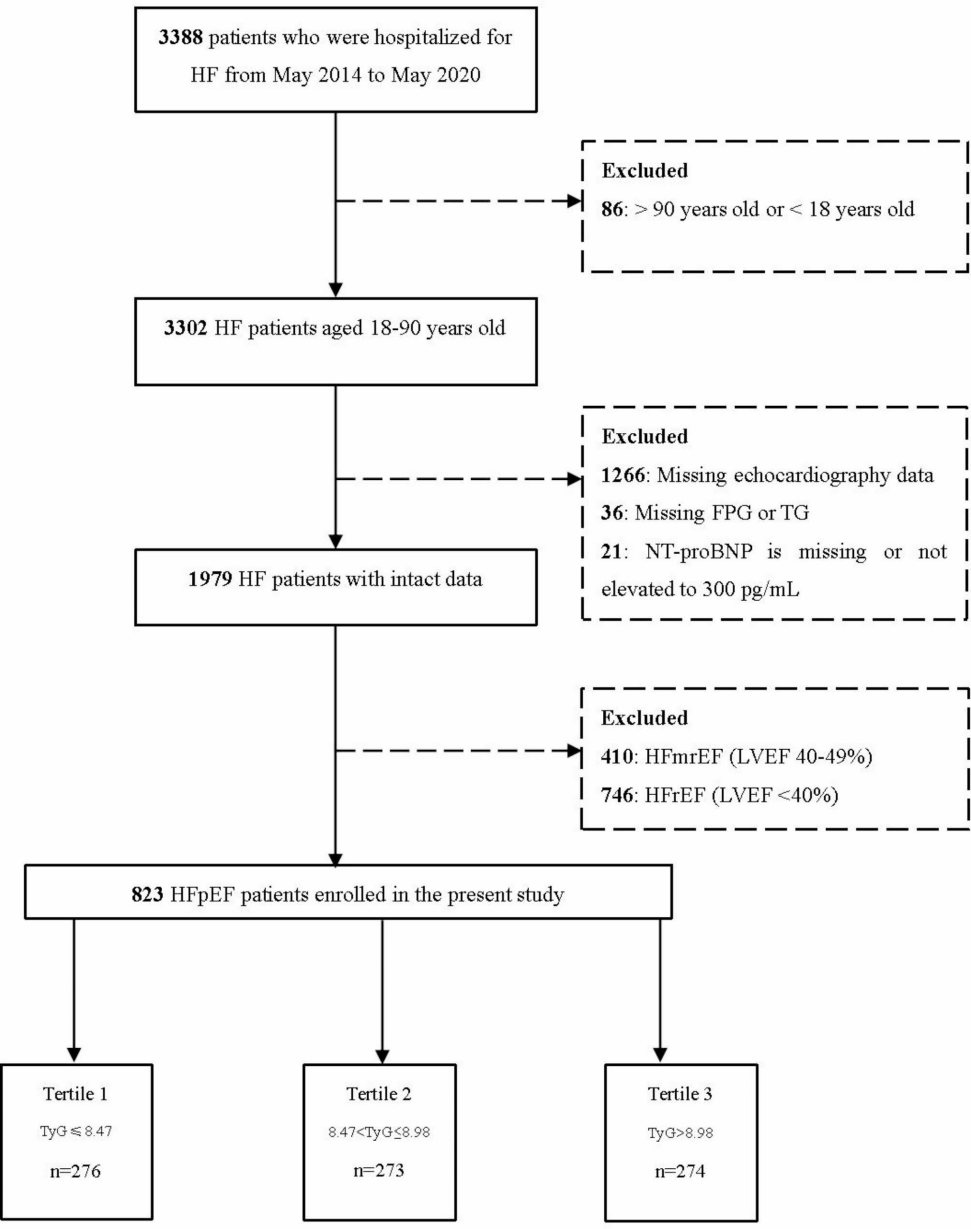
Flowchart of study population enrollment. Abbreviations: HF, heart failure; FPG, fasting plasma glucose; TG, triglyceride; NT-proBNP, N-terminal pro-B-type natriuretic peptide; LVEF, left ventricular ejection fraction; TyG, triglyceride-glucose; HFrEF, heart failure with reduced ejection fraction; HFmrEF, heart failure with mid-range ejection fraction; HFpEF, heart failure with preserved ejection fraction

This prospective study was conducted at the Peking University Third Hospital. A total of 3,388 patients hospitalized for acute heart failure (HF) between 2014 and 2020 were screened consecutively for eligibility. The diagnostic criteria of HFpEF were as follows: (1) left ventricular ejection fraction (LVEF) ≥ 50%; (2) typical heart failure symptoms and signs: breathlessness, coughing up pink frothy sputum, lower limb swelling or jugular venous distension; (3) N-terminal pro-B-type natriuretic peptide (NT-proBNP) levels exceeding 300 pg/mL; (4) relevant structural heart changes suggested by echocardiography (meeting any one of the following criteria): (a) left ventricular mass index > 115 g/m^2^ for males or > 95 g/m^2^ for females. (b)left atrial (LA) diameter > 40 mm for males or > 38 mm for females. (c) E/e’ ratio ≥ 9. The inclusion criteria were: (1) diagnosed with HFpEF; (2) aged 18–90 years; The exclusion criteria were as follows: (1) aged > 90 years or < 18 years; (2) missing echocardiography, FPG, TG, or NT-proBNP data. The detailed population enrollment process is presented in Fig. 1. During the study, patients were followed-up every six months after discharge through telephone calls or outpatient clinical visits. The patients were then categorized into tertiles based on their admission TyG index levels: the 1st tertile consisted of 276 patients with a TyG index ≤ 8.47, the 2nd tertile included 273 patients with a TyG index ranging 8.48–8.98, and the 3rd tertile comprised 274 patients with a TyG index > 8.98. The study was conducted in accordance with the principles of the Declaration of Helsinki and approved by the Ethics Review Committee of Peking University Third Hospital. Informed consent was obtained from all the participants.

### Data collection and endpoint definitions

Baseline demographic and clinical data, including age, sex, BMI, smoking history, laboratory tests, vital signs, echocardiographic data, comorbidities, and medication history, were retrieved from the electronic medical recording system. Peripheral venous blood samples were obtained from the participants within 24 h of hospital admission after an overnight fasting period of > 8 h. Laboratory tests included measurements of FPG, HbA1c, LDL-cholesterol (LDL-C), total cholesterol (TC), HDL-cholesterol (HDL-C), TG, NT-proBNP, creatinine (Cr), Hb, troponin T, and thyroid function. Medication history was also recorded, including the use of insulin, metformin, sodium-glucose cotransporter-2 inhibitors (SGLT2i), other hypoglycemic drugs, ACE inhibitors (ACEI), angiotensin receptor blockers (ARB), angiotensin receptor II blocker-neprilysin inhibitor (ARNI), calcium channel blockers (CCB), aldosterone receptor antagonists (MRA), and loop and thiazide diuretics.

BMI was defined as weight (kg) divided by height squared (m^2^). Diabetes was defined by fasting plasma glucose ≥ 126 mg/dL, a 2-hour oral glucose tolerance test value ≥ 200 mg/dL, HbA1c ≥ 6.5%, or random plasma glucose ≥ 200 mg/dL with classic symptoms of hyperglycemia. Hypertension was identified by a systolic blood pressure (SBP) ≥ 140 mmHg or a diastolic blood pressure (DBP) ≥ 90 mmHg, and/or the current use of antihypertensive medication. Hyperlipidemia was diagnosed when patients had LDL-C levels ≥ 140 mg/dL or a TG level ≥ 150 mg/dL.

The primary endpoint of this study was all-cause mortality. The secondary endpoints included CV death and rehospitalization due to HF.

### TyG index formula

The TyG index was calculated using the following formula: TyG index = ln(fasting TG [mg/dL] × FPG [mg/dL]/2).

### Statistical analyses

Continuous variables with a normal distribution were reported as mean ± standard deviation, while non-normally distributed variables were presented as median and interquartile range (IQR). Categorical variables were expressed as frequencies and percentages. Differences in continuous variables were assessed using analysis of variance (ANOVA) or the Kruskal–Wallis H test, whereas differences in categorical variables were evaluated using the chi-square test or Fisher’s exact test. We compared the baseline characteristics between the three TyG index groups of patients with HFpEF. We also compared the TyG index levels in patients with HFpEF (LVEF ≥ 50%) with those with heart failure with reduced ejection fraction (HFrEF) (LVEF < 40%) and those with heart failure with mid-range ejection fraction (HFmrEF) (LVEF 40–49%). In addition, we conducted a partial correlation analysis to assess the relationship between the TyG index and HFA-PEFF as well as H_2_FPEF scores, while controlling for age, smoking and coronary heart disease (CHD).

To explore the differences in event-free survival among the different TyG index groups in patients with HFpEF, Kaplan–Meier (K–M) survival analyses were performed. The time to event in this study was defined as the number of days that elapsed from the date of the blood draw to the occurrence of the endpoint or the censored date. Multivariate Cox regression analysis was conducted to examine the association between the TyG index and the incidence rates of the three outcomes. Variables that displayed notable differences among the TyG index tertiles, along with those of significant clinical relevance, were chosen as potential covariates for inclusion in the multivariable Cox regression model. A correlation matrix for these variables was generated, and a variance inflation factor (VIF) analysis was carried out. Variables with correlation coefficients greater than 0.6 or VIF values surpassing 10 were omitted from the Cox regression models. Finally, three models were used for adjustment: model 1 adjusted for age and sex; model 2 adjusted for model 1 variables plus BMI, SBP, HbA1c, HDL-C, Cr, Hb, cTnT,NT-proBNP, free thyroxine (FT4), diabetes, hyperlipidemia, and atrial fibrillation; and model 3 adjusted for model 2 variables plus LVEF, septal e’, pulmonary artery systolic pressure (PASP), tricuspid regurgitation (TR) velocity, LA diameter, LA pressure, right ventricular (RV) diameter and usage of insulin, metformin, SGLT2i and ACEI/ARB/ARNI. Tests for trends were performed by including the TyG index tertiles as ordinal variables in the statistical model, and significance was assessed using the Wald statistic. Several BMI values were missing. To effectively handle this issue, we employed the multiple imputation by chained equations (MICE) method, utilizing the “mice” function in R software. A competing risk analysis for HF rehospitalization was conducted, with all-cause death considered as the competing event. The Fine & Gray model was performed for this analysis. Furthermore, restricted cubic spline (RCS) analyses with four knots were conducted to examine the relationship between the TyG index and endpoints. Subgroup analyses were performed based on various factors, including age (< 65 or ≥ 65 years old), sex, BMI (< 25 or ≥ 25 kg/m²), cause of HFpEF (coronary heart disease [CHD] or others), diabetes, hyperlipidemia, renal dysfunction and LVEF (> 60% or 50–60%). To assess potential interactions, the regression models included cross-product terms between the TyG index and each potential effect modifier, and likelihood ratio tests were used to determine the significance of these interactions. To evaluate predictive performance, we utilized receiver operating characteristic (ROC) curves and calculated the area under the curve (AUC), comparing the Meta-Analysis Global Group in Chronic Heart Failure (MAGGIC) risk score [12] to the composite score of MAGGIC and the TyG index. The composite score of MAGGIC and TyG index = MAGGIC score + TyG index×2.

Statistical analyses were performed using SPSS Statistics (version 26; SPSS, Chicago, IL, USA) and R software (version 4.2.0). A two-tailed P-value < 0.05 was considered statistically significant.

## Results

### Baseline characteristics

A total of 823 patients diagnosed with HFpEF met the inclusion criteria and were enrolled in the study. The population had a mean age of 73.0 ± 12.7 years, comprising 427 (51.9%) female patients. Table 1 presents the baseline characteristics of the patients stratified by TyG index tertiles. A higher TyG index was associated with younger age; female sex; higher BMI; elevated Cr levels and SBP; a higher prevalence of diabetes, hyperlipidemia, and hypoglycemic drug use; and a lower proportion of atrial fibrillation. As shown in Supplemental Fig. 1, the TyG index was significantly lower in patients with HFpEF than in those with HFmrEF and HFrEF (P = 0.0005 and 0.0036, respectively). Among the patients with HFpEF, the partial correlation analysis revealed that, after controlling for age, smoking and CHD, the correlation between TyG index and HFA-PEFF score were insignificant (TyG index and HFA-PEFF score: correlation coefficient, 0.009, P = 0.801), as did the correlation between TyG index and H_2_FPEF score (TyG index and H_2_FPEF score: correlation coefficient, 0.002, P = 0.960).

**Table 1:** Baseline characteristics of the HFpEF patients according to TyG tertiles

Characteristic	Total	Tertile 1	Tertile 2	Tertile 3	*P* value
	n = 823	n = 276	n = 273	n = 274	
		TyG ≤ 8.47	8.47 < TyG ≤ 8.98	TyG > 8.98	
age, years	73.0 ± 12.7	75.2 ± 12.3	72.7 ± 12.5	71.1 ± 13.0	0.001*
Sex, female, n (%)	427 (51.9)	129 (46.7)	139 (50.9)	159 (58.0)	0.028*
NYHA class, n (%)					
I	81 (9.84)	24 (8.70)	26 (9.52)	31 (11.3)	0.387
II	398 (48.4)	136 (49.3)	127 (46.5)	135 (49.3)	
III	237 (28.8)	77 (27.9)	91 (33.3)	69 (25.2)	
IV	107 (13.0)	39 (14.1)	29 (10.6)	39 (14.2)	
BMI, kg/m2	25.5 ± 4.7	24.6 ± 4.6	25.4 ± 4.9	26.5 ± 4.5	< 0.001*
smoking, n (%)	188 (22.9)	58 (21.1)	60 (22.0)	70 (25.6)	0.406
Laboratory test					
FPG, mmol/L	6.20 [5.20, 7.90]	5.20 [4.60, 5.90]	6.10 [5.40, 7.20]	8.30 [6.60, 11.2]	< 0.001*
HbA1c, %	6.30 [5.80, 7.10]	6.00 [5.70, 6.40]	6.30 [5.80, 6.90]	7.00 [6.20, 8.20]	< 0.001*
NT-proBNP, pg/ml	1811 [840, 3704]	1888 [934, 3898]	1733 [828, 3637]	1794 [766, 3558]	0.493
LDL-C, mmol/L	2.22 ± 0.83	1.97 ± 0.72	2.25 ± 0.81	2.43 ± 0.89	< 0.001*
TC, mmol/L	3.75 ± 1.04	3.43 ± 0.85	3.72 ± 0.99	4.10 ± 1.16	< 0.001*
HDL-C, mmol/L	0.97 ± 0.29	1.06 ± 0.31	0.97 ± 0.27	0.88 ± 0.26	< 0.001*
TG, mmol/L	1.13 [0.84, 1.56]	0.77 [0.65, 0.92]	1.19 [1.01, 1.36]	1.81 [1.38, 2.29]	< 0.001*
Cr, µmol/L	98.0 [79.0, 134.0]	93.5 [76.0, 125.0]	97.0 [77.0, 126.0]	105.0 [83.0, 150.0]	0.002*
HGB, g/L	118 ± 23	116 ± 22	121 ± 24	118 ± 23	0.042*
cTnT, ng/mL	0.03 [0.02, 0.06]	0.03 [0.01, 0.05]	0.03 [0.02, 0.05]	0.04 [0.02, 0.09]	0.008*
TSH, mIU/L	1.87 [1.13, 3.16]	1.81 [1.12, 3.32]	1.82 [1.18, 3.13]	1.91 [1.04, 3.11]	0.673
FT3, pg/mL	2.62 ± 0.81	2.66 ± 1.07	2.65 ± 0.64	2.54 ± 0.64	0.175
FT4, ng/dL	1.25 [1.11, 1.43]	1.24 [1.11, 1.39]	1.30 [1.13, 1.47]	1.22 [1.07, 1.37]	0.004*
Vital signs					
Heart rate, beats/min	86 [76, 102]	84 [73, 98]	88 [76, 102]	89.5 [80, 110]	0.051
SBP, mmHg	138 ± 22	134 ± 22	139 ± 20	141 ± 23	< 0.001*
DBP, mmHg	73 ± 13	72 ± 13	74 ± 13	74 ± 13	0.057
Comorbidity					
Diabetes, n (%)	346 (42.0)	67 (24.3)	102 (37.4)	177 (64.6)	< 0.001*
Hypertension, n (%)	640 (77.8)	204 (73.9)	217 (79.5)	219 (79.9)	0.167
Coronary heart disease, n (%)	414 (50.3)	128 (46.4)	134 (49.1)	152 (55.5)	0.091
Atrial fibrillation, n (%)	381 (46.3)	152 (55.1)	128 (46.9)	101 (36.9)	< 0.001*
Hyperlipidemia, n (%)	266 (32.3)	67 (24.3)	91 (33.3)	108 (39.4)	0.001*
Hyperthyroid, n (%)	49 (6.19)	18 (6.79)	15 (5.77)	16 (5.99)	0.877
Renal dysfunction, n (%)	116 (14.1)	30 (10.9)	42 (15.4)	44 (16.1)	0.164
Medication use					
Insulin, n (%)	169 (20.5)	31 (11.2)	44 (16.1)	94 (34.3)	< 0.001*
SGLT2i, n (%)	116 (14.1)	21 (7.61)	42 (15.4)	53 (19.3)	< 0.001*
Metformin, n (%)	77 (9.36)	13 (4.71)	23 (8.42)	41 (15.0)	< 0.001*
ACEI/ARB/ARNI, n (%)	358 (43.5)	98 (35.5)	133 (48.7)	127 (46.4)	0.004*
Beta blocker, n (%)	435 (52.9)	141 (51.1)	148 (54.2)	146 (53.3)	0.753
MRA, n (%)	119 (14.5)	47 (17.0)	37 (13.6)	35 (12.8)	0.319
Loop diuretics, n (%)	485 (58.9)	170 (61.6)	161 (59.0)	154 (56.2)	0.438
Echocardiography					
LVEF, %	64.6 ± 7.7	64.9 ± 7.4	64.8 ± 8.0	64.0 ± 7.7	0.331
Septal E/e’ ratio	10.0 [8.0, 14.0]	11.0 [8.0, 14.0]	10.0 [8.0, 15.0]	10.0 [8.0, 14.0]	0.104
Septal e’, cm/s	8.85 ± 2.98	9.37 ± 3.30	8.91 ± 2.90	8.26 ± 2.61	< 0.001*
LV mass index, g/m^2^	100.0 ± 36.7	98.6 ± 36.9	102.0 ± 37.8	101.0 ± 35.4	0.534
LV wall thickness, mm	8.92 ± 1.60	8.90 ± 1.45	8.98 ± 1.67	8.89 ± 1.67	0.782
LVEDD, mm	48.9 ± 6.5	48.9 ± 7.1	48.9 ± 6.2	48.9 ± 6.3	0.999
TR velocity, m/s	2.78 ± 0.54	2.86 ± 0.55	2.82 ± 0.53	2.62 ± 0.52	< 0.001*
RWT	0.37 ± 0.08	0.37 ± 0.08	0.37 ± 0.08	0.37 ± 0.08	0.913
PASP, mmHg	38.0 ± 13.9	40.3 ± 15.1	38.6 ± 13.2	34.0 ± 12.0	< 0.001*
LA diameter, mm	41.3 ± 7.16	43.8 ± 8.59	40.7 ± 6.46	39.4 ± 5.45	< 0.001*
LA pressure, mmHg	15.0 ± 5.99	15.2 ± 5.52	15.0 ± 6.73	14.8 ± 5.66	0.855
RV diameter, mm	21.2 ± 4.17	22.6 ± 5.25	20.9 ± 3.58	20.3 ± 3.11	< 0.001*

Data are expressed as mean ± standard deviation, median [interquartile range] or n (%)

* refers to p < 0.05

ACEI: angiotensin-converting enzyme inhibitors; ARB: angiotensin receptor blockers; ARNI: angiotensin receptor II blocker - neprilysin inhibitor; BMI: body mass index; CCB: calcium channel blockers; Cr: creatinine; cTnT: troponin T; DBP: diastolic blood pressure; FPG, fasting plasma glucose; FT3: free triiodothyronine; FT4: free thyroxine; HDL-C: high-density lipoprotein cholesterol; HGB: hemoglobin; LA, left atrial; LDL-C: low-density lipoprotein cholesterol; LVEDD: left ventricular end-diastolic dimension; LV, left ventricular; LVEF: left ventricle ejection fraction; MRA: aldosterone receptor antagonist; NT-proBNP: N-terminal pro-B-type natriuretic peptide;NYHA, New York Heart Association; PASP, pulmonary artery systolic pressure; RV, right ventricular; RWT, relative wall thickness; SBP: systolic blood pressure; SGLT2i: sodium-glucose cotransporter-2 inhibitors; TC: total cholesterol; TG: triglycerides; TR, tricuspid regurgitation; TSH: thyroid stimulating hormone

### TyG index and outcomes

**Fig. 2:**
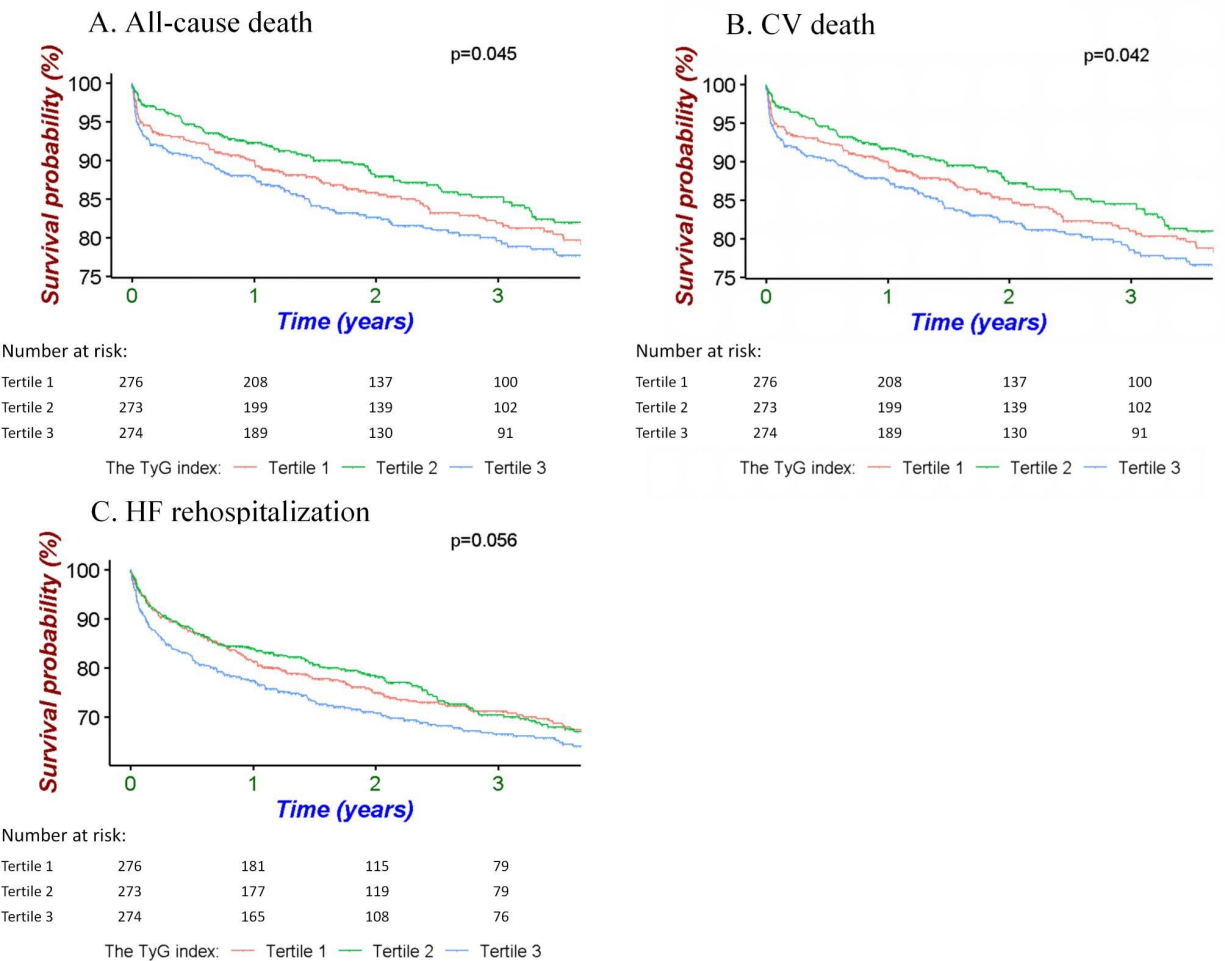
Kaplan–Meier analyses for different endpoints among the TyG index tertiles. A: all-cause death. B: CV death. C: HF rehospitalization. TyG, triglyceride-glucose; CV, cardiovascular; HF, heart failure

**Fig. 3:**
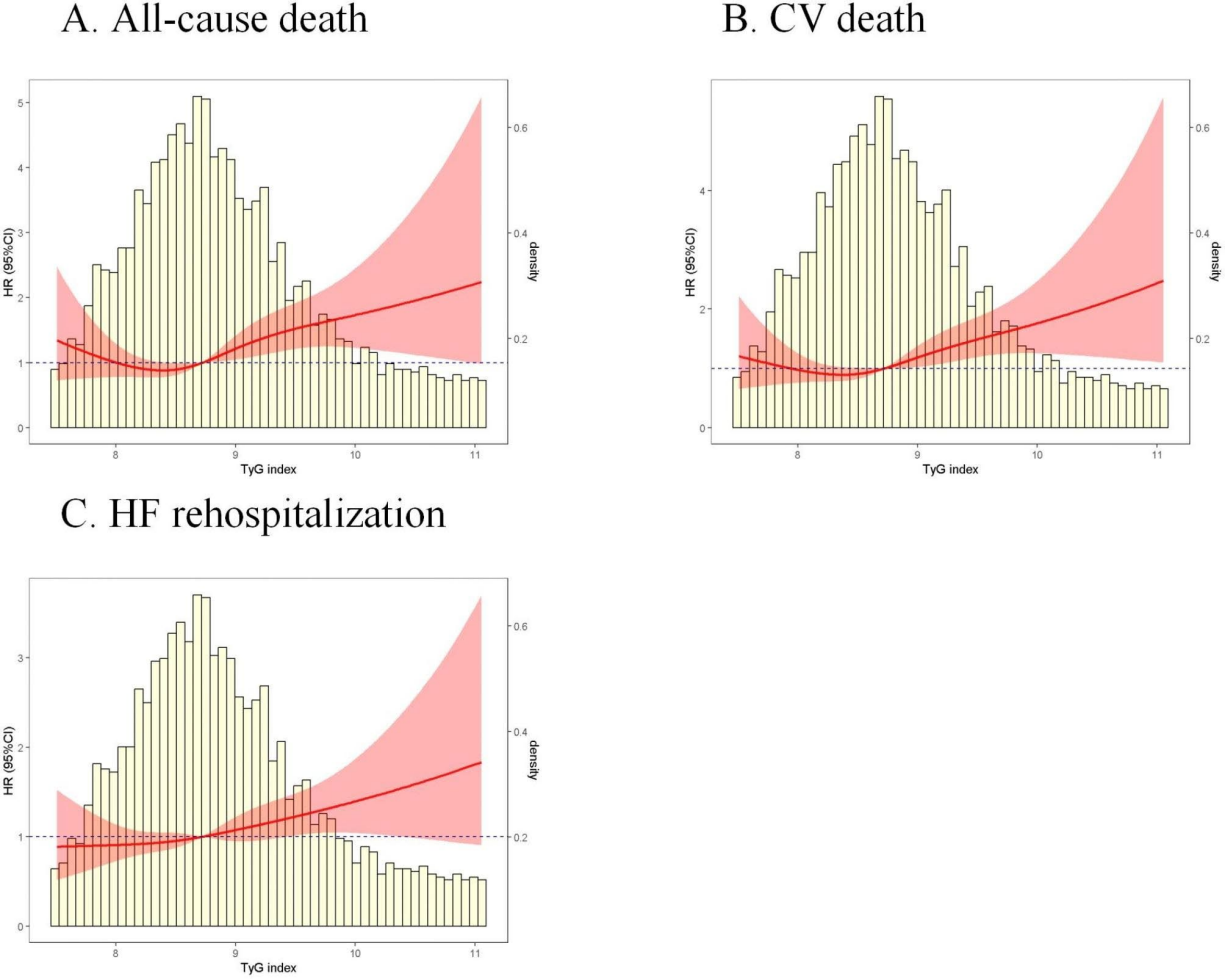
Nonlinear associations of the TyG index with different outcomes in the HFpEF patients. A: all-cause death. B: CV death. C: HF rehospitalization. TyG, triglyceride-glucose; HFpEF, heart failure with preserved ejection fraction; CV, cardiovascular; HF, heart failure

During a median follow-up of 3.16 years (IQR: 2.87–3.45 years), a total of 147 deaths (17.9% of the cohort) were recorded. K–M curves demonstrated that patients in the 3rd tertile had the lowest survival rate (p = 0.045; Fig. 2A). RCS analysis (Fig. 3A) revealed a J-shaped association between the TyG index and the risk of all-cause death. Table 2 presents the three multivariate Cox regression models used to assess the correlations between the TyG index and outcomes. In all three models, the highest TyG index tertile was associated with a higher incidence of all-cause death (model 1: HR 1.44, 95% CI 1.10–1.90, P = 0.009; model 2: HR 1.45, 95% CI 1.12–1.86, P = 0.004; model 3: HR 1.53, 95% CI 1.19–1.98, P < 0.001). The TyG index as a continuous variable also had significant association with all-cause death (Model 1: HR 1.41, 95% CI 1.06–1.88, P = 0.018; Model 2: HR 1.45, 95% CI 1.10–1.92, P = 0.008; Model 3: HR 1.46, 95% CI 1.10–1.96, P = 0.009). In the subgroup analyses, the TyG index was associated with a high incidence of all-cause mortality, which was consistent across subgroups including age, gender, BMI, CHD, diabetes, hyperlipidemia, renal dysfunction and LVEF (Supplemental Table 1). Meanwhile, there were no interactions between the TyG index and the variables in subgroup analyses (All P values for interaction > 0.05). Additionally, the ROC analysis demonstrated that the composite score of MAGGIC and TyG index (AUC 0.647, 95% CI 0.598–0.695) displayed superior predictive accuracy compared to MAGGIC alone (AUC 0.633, 95% CI 0.596–0.694, Supplemental Fig. 2). The net reclassification index (NRI) was 0.250 (95% CI 0.054–0.446, P = 0.012).

**Table 2:** Hazard ratios of triglyceride-glucose index tertiles for outcomes in the study participants

		Model 1				Model 2				Model 3		
Outcomes	Variables	HR	95%CI	*P-*value		HR	95%CI	*P-*value		HR	95%CI	*P-*value
All-cause death	TyG index*	1.41	1.06–1.88	0.018		1.45	1.10–1.92	0.008		1.46	1.10–1.96	0.009
	Tertile 1	Reference				Reference				Reference		
	Tertile 2	0.90	0.68–1.19	0.452		0.92	0.71–1.20	0.550		1.00	0.77–1.30	0.969
	Tertile 3	1.44	1.10–1.90	0.009		1.45	1.12–1.86	0.004		1.53	1.19–1.98	< 0.001
	*P* for trend			0.011				0.029				0.038
CV death	TyG index*	1.43	1.08–1.89	0.010		1.43	1.07–1.90	0.016		1.49	1.13–1.97	0.005
	Tertile 1	Reference				Reference				Reference		
	Tertile 2	0.92	0.7–1.22	0.576		0.94	0.72–1.21	0.612		1.01	0.79–1.32	0.880
	Tertile 3	1.45	1.10–1.90	0.007		1.44	1.12–1.85	0.004		1.52	1.19–1.96	< 0.001
	*P* for trend			0.009				0.042				0.045
HF rehospitalization	TyG index*	1.30	1.03–1.63	0.021		1.28	1.02–1.61	0.031		1.29	0.97–1.72	0.071
	Tertile 1	Reference				Reference				Reference		
	Tertile 2	1.00	0.79–1.26	0.981		0.95	0.77–1.18	0.681		1.07	0.77–1.49	0.698
	Tertile 3	1.34	1.06–1.70	0.013		1.34	1.09–1.65	0.004		1.36	0.98–1.90	0.069
	*P* for trend			0.013				0.041				0.077

HR, hazard ratio; CI, confidence interval; TyG, triglyceride glucose index; * TyG index as a continuous variable

Model 1 adjusted for age and sex

Model 2 adjusted for model 1 variables plus BMI, SBP, HbA1c, HDL-C, Cr, Hb, cTnT,NT-proBNP, FT4, diabetes, hyperlipidemia, and atrial fibrillation

Model 3 adjusted for model 2 variables plus LVEF, septal e’, PASP, TR velocity, LA diameter, LA pressure, RV diameter, and usage of insulin, metformin, SGLT2i and ACEI/ARB/ARNI

**Table 3:** Regression coefficients in competing risk model for heart failure rehospitalization

Variables	β	P	SHR	95% CI
TyG index*	0.154	0.031	1.17	1.02–1.34
Tertile 1	Reference			
Tertile 2	-0.049	0.650	0.95	0.77–1.17
Tertile 3	0.272	0.008	1.31	1.07–1.61

SHR, subdistribution hazard ratio; * TyG index as a continuous variable

The results were adjusted for age, sex, BMI, SBP, HbA1c, HDL-C, Cr, Hb, cTnT, NT-proBNP, FT4, diabetes, hyperlipidemia, atrial fibrillation, LVEF, septal e’, PASP, TR velocity, LA diameter, LA pressure, RV diameter and usage of insulin, metformin, SGLT2i and ACEI/ARB/ARNI

For CV death, a total of 139 deaths were recorded. The K–M curve also demonstrated that the 3rd tertile had a significantly lower event-free survival rate (Fig. 2B). Figure 3B shows a clear J-shaped association between the TyG index and CV death. In comparison to patients in the 1st tertile, those in the 3rd tertile exhibited a significantly higher risk of CV death (model 1: HR 1.45, 95% CI 1.10–1.90, P = 0.007; model 2: HR 1.44, 95% CI 1.12–1.85, P = 0.004; model 3: HR 1.52, 95% CI 1.19–1.96, P = 0.001). When treated as a continuous variable, the TyG index also demonstrated a significant association with CV mortality (Model 1: HR 1.43, 95% CI 1.08–1.89, P = 0.010; Model 2: HR 1.43, 95% CI 1.07–1.90, P = 0.016; Model 3: HR 1.49, 95% CI 1.13–1.97, P = 0.005). The subgroup analysis showed that the associations of TyG index with the risk of CV mortality was consistent across subgroups of age, sex, BMI, CHD, diabetes, renal dysfunction and LVEF. However, this association became insignificant in patients with dyslipidemia (Supplemental Table 2).

Regarding HF rehospitalization, 222 cases were recorded, accounting for 27.0% of the cohort. The K–M curve indicated that the 3rd tertile had the lowest event-free survival rate, whereas the curves for the 1st and 2nd tertiles exhibited some overlap (Fig. 2C). The RCS curve initially remained constant, and then rapidly increased (Fig. 3C). In the multivariable Cox regression analysis, compared to patients in the 1st tertile of the TyG index, those in the 3rd tertile had a significantly higher rate of HF rehospitalization in models 1 and 2, but this relationship became insignificant in model 3 (model 1: HR 1.34, 95% CI 1.06–1.70, P = 0.013; model 2: HR 1.34, 95% CI 1.09–1.65, P = 0.004; model 3: HR 1.36, 95% CI 0.98–1.90, P = 0.069). Similar results were observed when using the TyG index as a continuous value (Model 1: HR 1.30, 95% CI 1.03–1.63, P = 0.021; Model 2: HR 1.28, 95% CI 1.02–1.61, P = 0.031; Model 3: HR 1.29, 95% CI 0.97–1.72, P = 0.071). In the competing risk model shown in Table 3, a significant association between the TyG index and HF rehospitalization was observed. Specifically, when considering TyG as a continuous variable, the sub-distribution hazard ratio (SHR) was 1.17 (95% CI, 1.02–1.34, P = 0.031). When comparing TyG index tertile 3 to tertile 1, the SHR was 1.31 (95% CI, 1.07–1.61, P = 0.008). Subgroup analyses indicated that sex, BMI, CHD, hyperlipidemia, and renal dysfunction influenced this association. The association remained significant in the male, BMI ≥ 25 kg/m², CHD, non-hyperlipidemia, and non-renal dysfunction patients (Supplemental Table 3). The number of events in each tertile were presented in Supplemental Table 4.

## Discussion

To the best of our knowledge, this is the first study to investigate the relationship between TyG index and long-term prognosis in patients with HFpEF. Our findings demonstrate that an increased TyG index is an independent predictor of higher mortality and rehospitalization rates in patients with HFpEF.

Previous studies have examined the association between IR and HF. A prospective cohort study revealed that IR assessed using the hyperinsulinemic-euglycemic clamp technique can independently predict HF development [13]. However, that technique, regarded as the gold standard for evaluating insulin sensitivity, is invasive and costly, making it impractical in routine clinical practice. As an alternative, the Homeostatic Model Assessment for Insulin Resistance (HOMA-IR) has been widely used to assess IR, using fasting glucose and insulin levels. A meta-analysis reported that higher levels of IR assessed by HOMA-IR are associated with an increased risk of developing HF, even after accounting for traditional risk factors [14]. This association has also been observed in studies involving individuals with and without diabetes mellitus [14]. Another study demonstrated an inverse correlation between HOMA2-IR (an updated version of HOMA-IR) and improvements in ejection fraction in patients with HFrEF [15]. Other methods for assessing IR include the Quantitative Insulin Sensitivity Check Index (QUICKI) and Fasting Insulin Resistance Index (FIRI). In a study that used HOMA-IR, FIRI, and QUICKI to evaluate insulin sensitivity, all three assessment methods indicated impaired insulin sensitivity in patients with chronic HF compared with healthy controls [16].

However, owing to the complex mathematical calculations and the need for insulin concentration measurements, the clinical adoption of QUICKI, FIRI, and HOMA-IR for evaluating IR remains challenging. In the past decade, the TyG index has emerged as a simple and reliable surrogate marker of IR [17]. Previous studies have demonstrated a strong correlation between the TyG index and the gold-standard hyperinsulinemic-euglycemic clamp technique in individuals with and without diabetes. Moreover, compared to HOMA-IR, TyG index showed better evaluation efficiency. An advantage of the TyG index is that it relies on routine and low-cost biochemical tests, including serum triglycerides and glucose, making it easily accessible in any clinical setting. Recent studies have highlighted the association between the TyG index and risk of HF. A study based on two large cohorts and Mendelian randomization analysis reported that participants in the highest quartile of the TyG index had the highest risk of incident HF in both cohorts [18]. The results of the Mendelian randomization study revealed a causal relationship between a high TyG index and an increased risk of HF. Analyses of the Atherosclerosis Risk in Communities (ARIC) study have also shown that individuals in the highest quartile of the TyG index have a greater risk of incident HF and are more likely to experience adverse left ventricular remodeling and dysfunction [19]. Furthermore, several studies conducted in 2022 provided evidence to support the predictive value of the TyG index in patients with HF. Han et al. [20] found a significant association between an increased TyG index and in-hospital mortality among patients with HF, and Huang et al. [21]. reported that an elevated TyG index at admission was associated with higher mortality and major adverse cardiovascular and cerebrovascular event rates during a 478-day follow-up period in patients with HF. In the context of HFpEF, a recent cross-sectional study demonstrated a positive correlation between the TyG index and the risk of subclinical HFpEF in patients with type 2 diabetes mellitus [22]. Patients with a TyG index ≥ 9.47 showed an increased risk of metabolic syndrome and diastolic dysfunction. These studies collectively support the association between the TyG index and the incidence and prognosis of HF. However, data regarding the association between the TyG index and the long-term prognosis of HFpEF are still lacking, and the relationship between the TyG index and diastolic dysfunction remains unknown. Therefore, our study aimed to investigate the impact of IR, assessed using the TyG index, on the long-term prognosis of HFpEF.

Throughout the follow-up period, we observed a significant association between a high TyG index and an increased mortality rate. Additionally, in the competing risk model, which accounts for all-cause death as the competing event, we detected a higher rate of HF rehospitalization among individuals in the high TyG index tertiles. These findings imply that as time advanced during the three-year follow-up, patients with elevated TyG index levels experienced a heightened risk of mortality and more pronounced HF symptoms. Subgroup analyses revealed that this association was particularly prominent in patients with high BMI, CHD, and diabetes. This highlights the potential of the TyG index as an indicator of IR, especially in patients with metabolic syndromes. Furthermore, we compared the TyG index at baseline among patients with HFpEF, HFmrEF, and HFrEF and found that the TyG index was significantly lower in HFpEF patients than in those with HFmrEF and HFrEF. This finding suggests a positive association between the TyG index and severity of left ventricular systolic dysfunction. Additionally, we explored the relationship between the TyG index and the HFA-PEFF and H_2_FPEF scores among the 823 patients with HFpEF included in our study. We found that there was no significant association between the TyG index with HFA-PEFF and H_2_FPEF scores. In our study, patients with a high TyG index were typically younger, had higher BMI, were more likely to be smokers, and exhibited elevated levels of blood glucose and lipids. Additionally, a large proportion of these patients had CHD. These factors might elevate their risk of experiencing fatal myocardial infarction and stroke in the future. This may offer an explanation as to why individuals with a high TyG index experienced a poorer prognosis during the 3-year follow-up period.

The association between IR assessed using the TyG index and the prognosis of HFpEF can be explained by several mechanisms. First, a higher TyG index indicates IR, which is associated with metabolic abnormalities, such as dyslipidemia, hypertension, and obesity. These metabolic abnormalities can directly cause cardiovascular stiffening and contribute to the progression of HFpEF [23–25]. Secondly, the TyG index has been linked to endothelial dysfunction [26] and microvascular damage [27] characterized by impaired vasodilation and increased vascular resistance in small arteries and arterioles. In HFpEF, microvascular dysfunction plays a critical role in impairing the diastolic function by reducing myocardial perfusion and promoting myocardial fibrosis [28, 29]. Third, IR and metabolic dysfunction can trigger systemic inflammation, leading to increased levels of proinflammatory cytokines and activation of inflammatory pathways [30]. Inflammation then contributes to myocardial fibrosis, which is a hallmark of HFpEF [31].

Our findings revealed a significant association between higher TyG index levels and increased mortality and rehospitalization rates in patients with HFpEF over a 3-year follow-up period. The TyG index has been validated against established measures of IR, such as the hyperinsulinemic-euglycemic clamp [17] and HOMA-IR [32, 33]. Notably, it only requires fasting triglyceride and glucose measurements, which are readily accessible. Therefore, incorporating the TyG index into risk stratification protocols may facilitate the identification of individuals with HFpEF at high risk of death. Moreover, certain therapeutic interventions have demonstrated effectiveness in improving IR and reducing cardiac remodeling in patients with HF. For example, SGLT2i has shown promise for enhancing the cardiac prognosis of patients with type 2 diabetes mellitus, partly by alleviating cardiac IR [34]. Additionally, metformin has been found to significantly reduce left ventricular hypertrophy in patients with IR [35]. Hence, utilizing the TyG index to identify IR may enable early identification of high-risk individuals who could benefit from targeted interventions.

Moreover, IR is closely related to obesity. Clinical studies have presented evidence that obesity is linked to an elevated risk of mortality in patients with HFpEF [36]. Patients with obesity often exhibit hypertriglyceridemia and elevated plasma levels of fatty acids. These lipids are absorbed and stored within lipid droplets in the heart [37]. When intramyocardial lipid accumulation exceeds the heart’s capacity for storage and oxidation, it can become lipotoxic, leading to the development of nonischemic and nonhypertensive cardiomyopathy, commonly referred to as diabetic cardiomyopathy or lipotoxic cardiomyopathy. The clinical characteristics of diabetic cardiomyopathy typically include cardiac hypertrophy and diastolic dysfunction, which culminate in HF, particularly HFpEF [38].

Nevertheless, our study has some limitations. First, owing to the unavailability of fasting insulin levels for most subjects, we were unable to compare the predictive power of different methods for assessing IR in our observational study. Second, we only measured the TyG index at baseline, precluding the assessment of its fluctuations during the follow-up period. Third, despite our best efforts to include a wide range of covariates and potential confounding factors in our analysis, there might still be unmeasured variables such as genetic factors, dietary patterns, psychosocial factors and health care disparities. Fourth, we were unable to capture long-term changes in diastolic function or monitor the incidence of myocardial infarction and stroke. Additionally, we were unable to monitor changes in the NYHA classification and BNP levels during the follow-up period.

## Conclusions

Our study demonstrated that increased IR, as indicated by the TyG index, is a significant predictor of all-cause and CV mortality in patients with HFpEF. The TyG index can serve as a valuable tool for identifying early insulin-resistant patients with HFpEF, enabling improved risk stratification and guiding subsequent interventions. These findings underscore the importance of considering IR in the management and prognosis of HFpEF.

### Electronic supplementary material

Below is the link to the electronic supplementary material.

Supplementary Material 1

## Data Availability

The datasets used and analyzed during the current study are available from the corresponding author upon reasonable request.

## Abbreviations

ACEI: Angiotensin-converting enzyme inhibitors
ARB: Angiotensin receptor blockers
ARNI: Angiotensin receptor II blocker - neprilysin inhibitor
BMI: Body mass index
CCB: Calcium channel blockers
CHD: Coronary heart disease
Cr: Creatinine
cTnT: Troponin T
CV: Cardiovascular
DBP: Diastolic blood pressure
E/e’: The Ratio of early diastolic mitral inflow velocity to septal mitral annulus tissue relaxation velocity in early diastole
FPG: Fasting plasma glucose
FT3: Free triiodothyronine
FT4: Free thyroxine
HDL-C: High-density lipoprotein cholesterol
HF: Heart failure
HFmrEF: Heart failure with mid-range ejection fraction
HFpEF: Heart failure with preserved ejection fraction
HFrEF: Heart failure with reduced ejection fraction
HGB: Hemoglobin
LDL-C: Low-density lipoprotein cholesterol
LVEDD: Left ventricular end-diastolic dimension
LVEF: Left ventricle ejection fraction
MRA: Aldosterone receptor antagonist
NT-proBNP: N-terminal pro-B-type natriuretic peptide
SBP: Systolic blood pressure
SGLT2i: Sodium-glucose cotransporter-2 inhibitors
TC: Total cholesterol
TG: Triglycerides
TSH: Thyroid stimulating hormone
TyG: Triglyceride-glucose index
