# Supplementary material for: High triglyceride-glucose (TyG) index is associated with poor prognosis of heart failure with preserved ejection fraction
Source: Cardiovasc Diabetol. 2023 Sep 29;22:263. doi: 10.1186/s12933-023-02001-4 (PMC10541699; doi:10.1186/s12933-023-02001-4)
Supplement: Supplementary file 1 — Supplementary Material 1 [file 12933_2023_2001_MOESM1_ESM.docx]

**Supplementary materials**

Supplement to: High triglyceride-glucose (TyG) index is associated with poor prognosis of heart failure with preserved ejection fraction

**Table of contents**

Supplementary Figure 1. Comparison of TyG index levels among HFpEF, HFmrEF and HFrEF

Supplementary Figure 2. ROC curves of MAGGIC risk score and the composite score of MAGGIC and TyG index

Supplementary Table 1. Subgroup analyses of the association between TyG index and all-cause death according to patient characteristics and comorbidities

Supplementary Table 2. Subgroup analyses of the association between TyG index and CV death according to patient characteristics and comorbidities

Supplementary Table 3. Subgroup analyses of the association between TyG index and HF rehospitalization according to patient characteristics and comorbidities

Supplementary Table 4. The number of events among the three TyG index groups


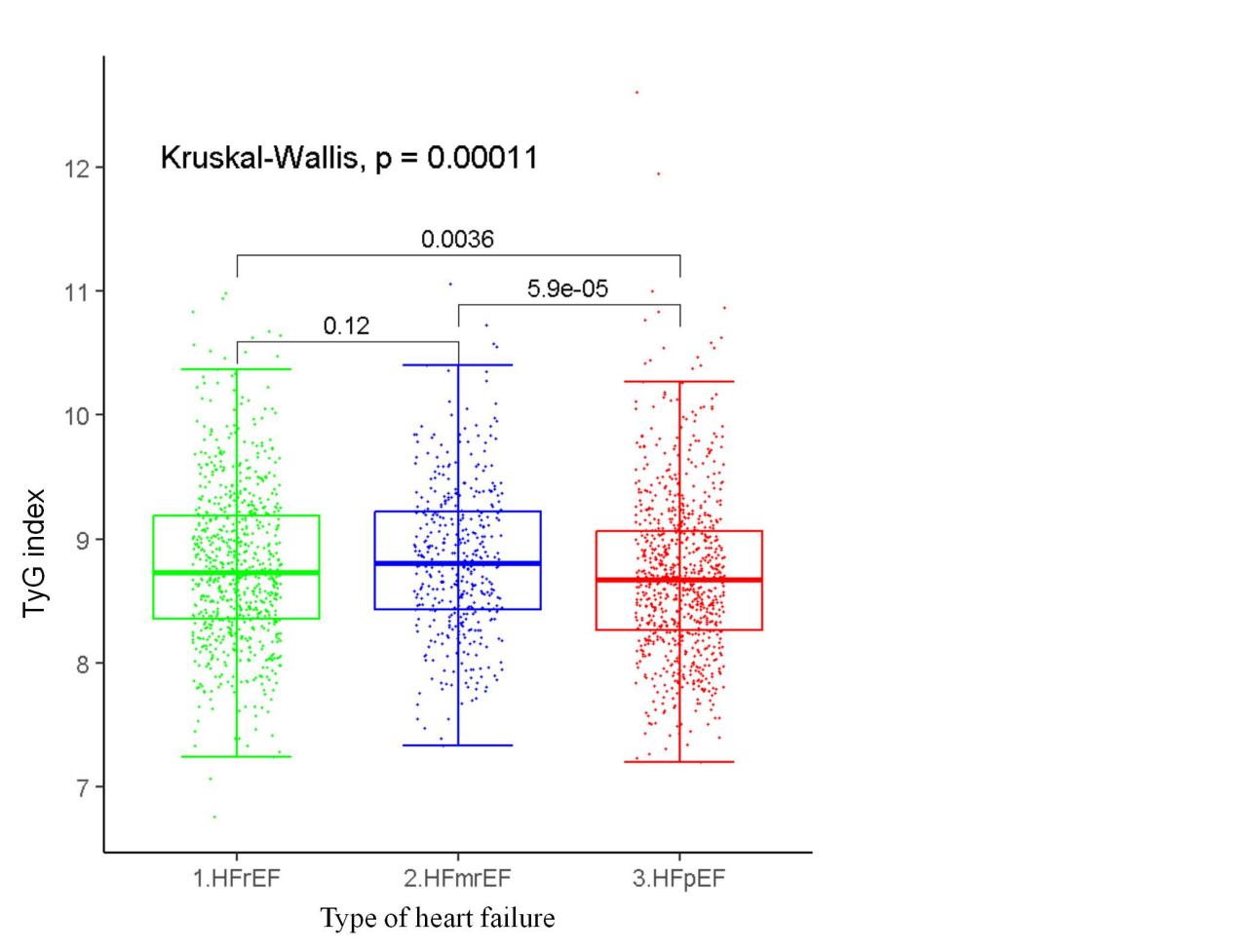


Supplementary Figure 1. Comparison of TyG index levels among HFpEF, HFmrEF and HFrEF.

Abbreviations: TyG, triglyceride-glucose; HFrEF, heart failure with reduced ejection fraction; HFmrEF, heart failure with mid-range ejection fraction; HFpEF, heart failure with preserved ejection fraction.


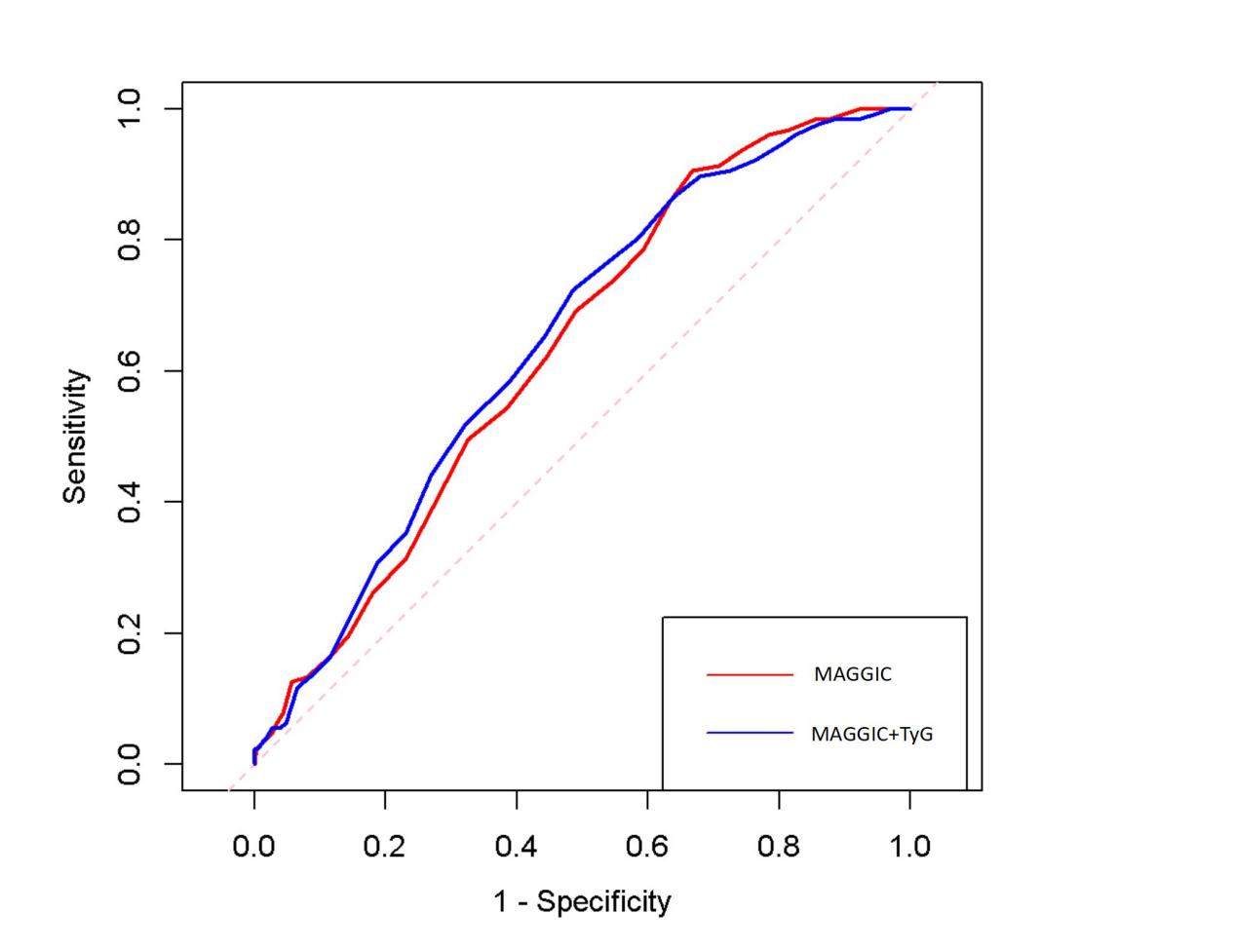


Supplementary Figure 2. ROC curves of MAGGIC risk score and the composite score of MAGGIC and TyG index.

Abbreviations: TyG, triglyceride-glucose; MAGGIC, Meta-Analysis Global Group in Chronic Heart Failure

Supplementary Table 1. Subgroup analyses of the association between TyG index and all-cause death according to patients characteristics and comorbidities.

| Subgroup | Events/Total | HR | 95%CI | P-value | *P_interaction_* |
| --- | --- | --- | --- | --- | --- |
| **Age, year** |  |  |  |  |  |
| <65 | 17/188 | 1.30 | 1.01-1.66 | 0.042 | 0.754 |
| ≥65 | 130/635 | 1.56 | 1.07-2.28 | 0.022 |  |
| **Gender** |  |  |  |  |  |
| Female | 73/396 | 1.56 | 1.16-2.10 | 0.004 | 0.178 |
| Male | 74/427 | 1.26 | 1.01-1.58 | 0.045 |  |
| **BMI** |  |  |  |  |  |
| <25 kg/m^2^ | 132/694 | 1.40 | 1.06-1.84 | 0.017 | 0.306 |
| ≥25 kg/m^2^ | 15/129 | 1.42 | 1.02-2.09 | 0.040 |  |
| **Cause of HFpEF** |  |  |  |  |  |
| CHD | 75/414 | 1.43 | 1.08-1.89 | 0.012 | 0.977 |
| Other causes | 72/409 | 1.27 | 1.01-1.60 | 0.045 |  |
| **Diabetes** |  |  |  |  |  |
| Yes | 68/346 | 1.53 | 1.09-2.17 | 0.017 | 0.589 |
| No | 79/477 | 1.36 | 1.01-1.85 | 0.049 |  |
| **Hyperlipidemia** |  |  |  |  |  |
| Yes | 34/266 | 1.67 | 1.03-2.72 | 0.040 | 0.653 |
| No | 113/557 | 1.49 | 1.23-1.81 | <0.001 |  |
| **Renal dysfuncion** |  |  |  |  |  |
| Yes | 31/116 | 1.33 | 1.10-1.61 | 0.003 | 0.450 |
| No | 116/707 | 1.53 | 1.09-2.17 | 0.017 |  |
| **LVEF** |  |  |  |  |  |
| >60% | 92/562 | 1.31 | 1.01-1.73 | 0.049 | 0.643 |
| 50%-60% | 55/261 | 1.60 | 1.02-2.48 | 0.037 |  |

HR and 95%CI was based on per 1-uint increase in TyG index.

HR, hazard ratio; CI, confidence interval; BMI, body mass index; HFpEF, heart failure with preserved ejection fraction; CHD, coronary heart disease.

Supplementary Table 2. Subgroup analyses of the association between TyG index and CV death according to patients characteristics and comorbidities.

| Subgroup | Events/Total | HR | 95%CI | P-value | *P_interaction_* |
| --- | --- | --- | --- | --- | --- |
| **Age, year** |  |  |  |  |  |
| <65 | 17/188 | 1.31 | 1.02-1.68 | 0.033 | 0.676 |
| ≥65 | 122/635 | 1.49 | 1.03-2.17 | 0.035 |  |
| **Gender** |  |  |  |  |  |
| Female | 71/427 | 1.48 | 1.03-2.12 | 0.033 | 0.193 |
| Male | 68/396 | 1.31 | 1.01-1.69 | 0.043 |  |
| **BMI** |  |  |  |  |  |
| <25 kg/m^2^ | 125/694 | 1.39 | 1.06-1.82 | 0.017 | 0.255 |
| ≥25 kg/m^2^ | 14/129 | 1.68 | 1.03-2.73 | 0.040 |  |
| **Cause of HFpEF** |  |  |  |  |  |
| CHD | 72/414 | 1.46 | 1.01-2.12 | 0.047 | 0.981 |
| Other causes | 67/409 | 1.29 | 1.04-1.59 | 0.020 |  |
| **Diabetes** |  |  |  |  |  |
| Yes | 66/346 | 1.70 | 1.05-2.75 | 0.034 | 0.462 |
| No | 73/477 | 1.36 | 1.01-1.84 | 0.045 |  |
| **Hyperlipidemia** |  |  |  |  |  |
| Yes | 31/266 | 0.97 | 0.65-1.45 | 0.893 | 0.771 |
| No | 108/557 | 1.51 | 1.18-1.93 | 0.001 |  |
| **Renal dysfuncion** |  |  |  |  |  |
| Yes | 28/116 | 1.38 | 1.02-1.87 | 0.038 | 0.491 |
| No | 111/707 | 1.38 | 1.09-1.76 | 0.008 |  |
| **LVEF** |  |  |  |  |  |
| >60% | 88/562 | 1.34 | 1.01-1.78 | 0.040 | 0.609 |
| 50%-60% | 51/261 | 1.55 | 1.02-2.35 | 0.041 |  |

HR and 95%CI was based on per 1-uint increase in TyG index.

HR, hazard ratio; CI, confidence interval; CV, cardiovascular; BMI, body mass index; HFpEF, heart failure with preserved ejection fraction; CHD, coronary heart disease.

Supplementary Table 3. Subgroup analyses of the association between TyG index and HF rehospitalization according to patients characteristics and comorbidities.

| Subgroup | Events/Total | HR | 95% CI | P-value | *P_interaction_* |
| --- | --- | --- | --- | --- | --- |
| **Age, year** |  |  |  |  |  |
| <65 | 41/188 | 1.36 | 1.03-1.78 | 0.030 | 0.845 |
| ≥65 | 181/635 | 1.29 | 1.04-1.59 | 0.020 |  |
| **Gender** |  |  |  |  |  |
| Female | 124/427 | 1.14 | 0.98-1.32 | 0.087 | 0.256 |
| Male | 98/396 | 1.15 | 1.00-1.32 | 0.045 |  |
| **BMI** |  |  |  |  |  |
| <25 kg/m^2^ | 191/694 | 1.19 | 0.90-1.59 | 0.225 | 0.278 |
| ≥25 kg/m^2^ | 31/129 | 1.30 | 1.05-1.60 | 0.015 |  |
| **Cause of HFpEF** |  |  |  |  |  |
| CHD | 113/414 | 1.04 | 1.01-1.07 | 0.017 | 0.798 |
| Other causes | 109/409 | 1.11 | 0.89-1.37 | 0.364 |  |
| **Diabetes** |  |  |  |  |  |
| Yes | 98/346 | 1.40 | 1.08-1.80 | 0.011 | 0.576 |
| No | 124/477 | 1.78 | 1.13-2.82 | 0.015 |  |
| **Hyperlipidemia** |  |  |  |  |  |
| Yes | 73/266 | 0.89 | 0.66-1.21 | 0.455 | 0.731 |
| No | 149/557 | 1.29 | 1.05-1.59 | 0.014 |  |
| **Renal dysfuncion** |  |  |  |  |  |
| Yes | 35/116 | 1.27 | 0.85-1.88 | 0.239 | 0.245 |
| No | 187/707 | 1.38 | 1.04-1.83 | 0.021 |  |
| **LVEF** |  |  |  |  |  |
| >60% | 151/562 | 1.18 | 0.95-1.46 | 0.123 | 0.376 |
| 50%-60% | 71/261 | 1.21 | 0.84-1.73 | 0.299 |  |

HR and 95%CI was based on per 1-uint increase in TyG index.

HR, hazard ratio; CI, confidence interval; BMI, body mass index; HF, heart failure; HFpEF, heart failure with preserved ejection fraction; CHD, coronary heart disease.

Supplementary Table 4. The number of events among the three TyG index groups.

| Endpoints | Overall n = 823 | Tertile 1 n = 276 | Tertile 2 n = 273 | Tertile 3 n = 274 |
| --- | --- | --- | --- | --- |
| All-cause death | 147 (17.9) | 52 (19.0) | 41 (15.0) | 54 (19.6) |
| CV death | 139 (16.9) | 50 (18.2) | 37 (13.6) | 52 (18.8) |
| HF rehospitalization | 222 (27.0) | 72 (26.1) | 70 (25.6) | 80 (29.2) |

The numbers of events are shown as n (%).

CV cardiovascular, HF heart failure
